# Investigating the role of endogenous estrogens, hormone replacement therapy, and blockade of estrogen receptor-α activity on breast metabolic signaling

**DOI:** 10.1007/s10549-021-06354-w

**Published:** 2021-08-26

**Authors:** Alana A. Arnone, J. Mark Cline, David R. Soto-Pantoja, Katherine L. Cook

**Affiliations:** 1 Department of Physiology and Pharmacology, Wake Forest University Health Sciences, Winston-Salem, NC 27157 USA; 2 Department of Surgery, Wake Forest School of Medicine, Winston-Salem, NC 27157 USA; 3 Department of Pathology, Section on Comparative Medicine, Wake Forest School of Medicine, Winston-Salem, NC 27157 USA; 4 Department of Cancer Biology, Wake Forest School of Medicine, Winston-Salem, NC 27157 USA; 5 Comprehensive Cancer Center, Wake Forest School of Medicine, Winston-Salem, NC 27157 USA

**Keywords:** Non-targeted metabolomics, Estrogen, Tamoxifen, Breast cancer, Post-menopausal, Hormone replacement therapies, Conjugated equine estrogen

## Abstract

**Purpose:**

Menopause is associated with an increased risk of estrogen receptor-positive (ER +) breast cancer. To characterize the metabolic shifts associated with reduced estrogen bioavailability on breast tissue, metabolomics was performed from ovary-intact and ovariectomized (OVX) female non-human primates (NHP). The effects of exogenous estrogen administration or estrogen receptor blockade (tamoxifen treatment) on menopause-induced metabolic changes were also investigated.

**Methods:**

Bilateral ovariectomies were performed on female cynomolgus macaques (*Macaca fascicularis*) to model menopause. OVX NHP were then divided into untreated (*n* = 13), conjugated equine estrogen (CEE)-treated (*n*= 13), or tamoxifen-treated (*n* = 13) subgroups and followed for 3 years. Aged-matched ovary-intact female NHP (*n* = 12) were used as a premenopausal comparison group. Metabolomics was performed on snap-frozen breast tissue.

**Results:**

Changes in several different metabolic biochemicals were noted, particularly in glucose and fatty acid metabolism. Specifically, glycolytic, Krebs cycle, acylcarnitines, and phospholipid metabolites were elevated in breast tissue from ovary-intact NHP and OVX + CEE in relation to the OVX and OVX + tamoxifen group. In contrast, treatment with CEE and tamoxifen decreased several cholesterol metabolites, compared to the ovary-intact and OVX NHP. These changes were accompanied by elevated bile acid metabolites in the ovary-intact group.

**Conclusion:**

Alterations in estrogen bioavailability are associated with changes in the mammary tissue metabolome, particularly in glucose and fatty acid metabolism. Changes in these pathways may represent a bioenergetic shift in gland metabolism at menopause that may affect breast cancer risk.

**Supplementary Information:**

The online version contains supplementary material available at 10.1007/s10549-021-06354-w.

## Introduction

Breast cancer is the most common form of cancer in the USA. Among women, breast cancer accounts for 30% of all cancer diagnoses, with 281,550 new cases estimated in 2021 [1]. Factors related to estrogen production are linked to an increased breast cancer risk, suggesting a mechanistic association between estrogen signaling and the development of breast carcinogenesis [2–5]. Early menarche, late menopause, obesity, or the use of hormone replacement therapies, which all increase lifetime exposure to estrogen, are associated with increased breast cancer risk in both pre-and post-menopausal women. [4, 6–8].

Circulating estrogen and postmenopausal breast cancer risk are linked in numerous studies [9–11]. The Endogenous Hormones and Breast Cancer Collaborative Group (EHBCCG) reanalyzed data from nine prospective studies on endogenous hormone levels and breast cancer risk in postmenopausal women. They found that levels of total estradiol, free estradiol, estrone, and estrone sulfate were associated with increased breast cancer risk. Specifically, postmenopausal women in the highest quintile of plasma free estradiol (E2) had a 2.58-fold (95% CI 1.76–3.78) higher rate of breast cancer over 10 years compared to women in the lowest quintile [10, 12]. Thomas and colleagues found that postmenopausal women who later developed breast cancer had 15% higher serum estradiol than women who remained breast cancer free [13].

Endogenous estradiol (E2) plays a fundamental role in controlling several metabolic pathways, including energy homeostasis, glucose metabolism, and nucleotide sugar metabolism [14]. Elevated glucose has been associated with increased breast cancer incidence [15–17]. Reprogramming of cancer metabolism is a recognized hallmark of malignancy. In general, cancer cells preferentially undergo glycolysis in an oxygen-rich environment, unlike normal cells that prefer oxidative phosphorylation [18]. Nucleotide sugar metabolism drives aberrant cell surface glycosylation, which supports cancer cell migration and signaling [19]. Additionally, estrogens regulate several enzymes in the tricarboxylic acid cycle (TCA) cycle, including the condensation reaction between acetyl-CoA and oxaloacetate to form citrate, which is catalyzed by citrate synthase, whose activity is enhanced by E2 [20].

Estrogens are essential modulators of lipid metabolism, particularly in the β-oxidation of fatty acids [21, 22]. However, cancer cells can alter aspects of lipid metabolism, including the availability of structural lipids for membrane synthesis and the synthesis of lipids for energy homeostasis. These changes in lipid metabolism can affect cell growth, proliferation, and differentiation. Carnitine system metabolites, which facilitate transport of fatty acids into mitochondria, are associated with breast cancer risk [23].

Furthermore, estrogens are implicated in controlling bile acid (BA) levels [24]. BA are synthesized in the liver from cholesterol [25]. Studies show BA have anti-carcinogenic effects in several cancer cell models, including breast cancer [26–28]. Specifically, lithocholic acid (LCA) exhibits anti-proliferative and pro-apoptotic effects in both MCF-7 and MDA-MB-231 breast cancer cells. Using metabolomics, Tang et al. showed that BA accumulate in the tumors of specific subsets of breast cancer. Tumors with increased BA showed a decrease in proliferation, suggesting an association with better patient survival. [29]

Hormonal replacement therapies (HRT), the main treatment for menopause-related symptoms, are implicated in breast cancer development [30, 31]. Estrogen-only HRT, such as conjugated equine estrogens (CEE), mimic endogenous estrogen’s effects. Numerous studies reported a link between HRT use and postmenopausal breast cancer risk [31, 32]. The Women’s Health Initiative (WHI) randomized 16,608 postmenopausal women with an intact uterus and ovaries to either placebo or a combination of 0.625 mg CEE and 2.5 mg MPA and 10,739 postmenopausal women who had undergone hysterectomies to either 0.625 mg CEE or placebo. After 5.6 years, the CEE + MPA group had a 24% increase in invasive breast cancer risk compared to the placebo group. In contrast, there was no increased risk of breast cancer in the CEE-only group [32, 33].

Adjuvant treatment of postmenopausal estrogen receptor-α (ER)+ breast cancer involves reducing estrogen secretion through endocrine-targeting therapies such as selective estrogen receptor modulators (SERM). Tamoxifen (TAM), a SERM, inhibits the expression of estrogen-regulated genes such as growth factors and angiogenic factors secreted by the tumor in breast tissue. Blockade of these genes results in a slowing of cell proliferation and tumor regression [34]. In other tissues, such as the uterus, TAM has an estrogenic effect, activating ER gene cofactors [35]. The adjuvant administration of TAM reduced the recurrence of breast cancer and prolonged survival in women with operable breast cancer by 47% and the risk of death by 26% in patients with hormone-receptor-positive breast cancer [36]. Additionally, TAM reduces the risk of invasive and non-invasive breast cancer by 49% and 50% respectively [37].

As with E2, HRT and endocrine therapies are associated with alterations in metabolism. Both CEE and tamoxifen affect cellular lipid metabolism. TAM also has similar effects on the protection of cell membranes as endogenous estrogens [38]. In postmenopausal women with early-stage breast cancer, TAM administration was associated with a favorable effect on lipid profiles [39]. CEEs are associated with favorable lipid profiles, with CEE administration reducing low-density lipoprotein cholesterol and increasing high-density lipoprotein cholesterol [40].

To characterize the metabolic shifts associated with estrogen bioavailability on breast metabolic pathways, metabolomics was performed on mammary gland tissue from ovariectomized female *Macaca fascicularis* monkeys. The effects of conjugated equine estrogen (CCE) or estrogen receptor-α antagonist (tamoxifen) treatment on modification of menopause-induced metabolic changes were studied. This primate species is a well-established model of women’s health, particularly for the study of breast cancer [41–43]. Specifically, ovariectomized NHP display low levels of circulating estrogens, which are physiologically relevant to postmenopausal women [44]. The present study demonstrates several metabolites strongly differentiate between treatment groups, suggesting that these metabolites may be associated with estrogen-dependent changes in the mammary tissue metabolome.

## Methods

Animals and study design. Methods were adapted from Cline et al. [44]. In brief, adult female cynomolgus macaques (*M. fascicularis)* were imported from Indonesia (Institut Pertanian Bogor or Charles River Primates, Port Washington, NY). Bilateral ovariectomies were performed on NHP 3 months before treatment. Ovariectomized NHP were untreated (control group) or continuously treated with either conjugated equine estrogens (CEE) or tamoxifen for 3 years. Treatment was administered in the diet at doses equivalent on a caloric basis to 0.625 mg/woman/ day for CEE and 20 mg/day for tamoxifen. To confirm the ovariectomy’s success, serum estradiol and progesterone were measured before treatment. Estradiol, estrone, and tamoxifen were measured throughout the study. The NHP’s age was determined at randomization by dentition, with the mean age of 7.5 years at the study’s end. NHP were housed in social groups of 4–6 monkeys in an AAALAC-accredited facility. NHP were fasted starting at 3 pm the day prior to euthanasia with free access to water. All experimental protocols were approved by the Institutional Animal Care and Use Committee. Aged-matched ovary-intact NHP were used as the endogenous estrogen group. At the end of the study, mammary tissue was removed and snap-frozen, and stored at − 80 °C.

Metabolomics analysis. Metabolomics was performed on mammary gland samples by Metabolon, Raleigh, NC as previously described [45–47]. Samples were prepared using the automated MicroLab Star system from the Hamilton Company. The extract was divided into five fractions: two for analysis by two separate reverse phase (RP)/UPLC-MS/MS with positive ion mode electrospray ionization (ESI), one for analysis by RP/UPLC-MS/MS with negative ion mode ESI, and one for analysis by HILIC/UPLC-MS/MS with negative mode ESI; one for backup.

Ultrahigh Performance Liquid Chromatography-Tandem Mass Spectroscopy (UPLC-MS/MS). A Waters ACQUITY ultra-performance liquid chromatography (UPLC) system and a Thermo Scientific Q-Exactive mass spectrometer interfaced with a heated electrospray ionization (HESI-II) source and Orbitrap mass analyzer was used. Compounds were identified by comparison to library entries of purified standards or recurrent unknown entities. Peaks were quantified using area-under-the-curve.

Metabolomics, bioinformatics, and statistics. The informatics system consisted of the Laboratory Information Management System (LIMS), the data extraction and peak-identification software, data processing tools for QC and compound identification, and a collection of information interpretation and visualization tools [48]. The hardware and software foundations for these informatics components were in a LAN backbone and a database server running Oracle 10.2.1.1 Enterprise Edition, respectively. Log transformation and imputation of missing values were performed with the minimum observed value for each compound. A Welch’s two-sample t-test was used to identify biochemicals that differed significantly between experimental groups. A total of 801 known biochemical compounds were identified. A p-value of *p* ≤ 0.05 was considered statistically significant. The numbers of biochemicals that reached statistical significance as well as those approaching significance (0.05 < *p* < 0.10) are outlined in Supplemental Table 1.

## Results

**Fig. 1:**
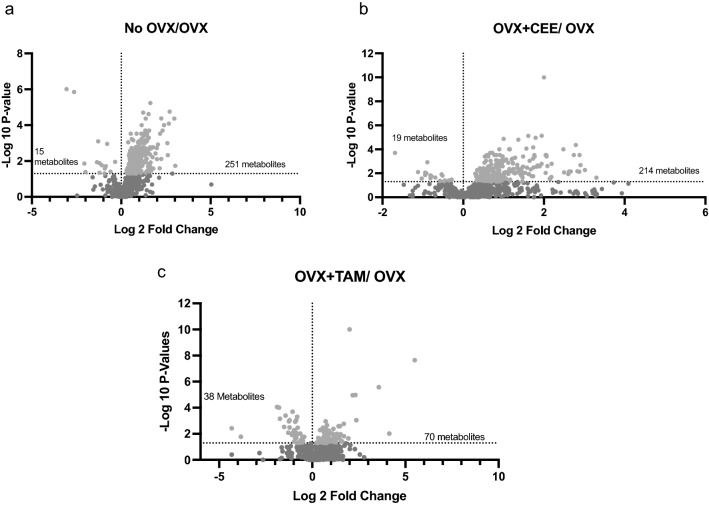
Volcano plots showing the distribution of metabolites by group. **a** Volcano map showing the distribution of metabolites in the ovary-intact (non-ovariectomized; no-OVX) vs ovariectomized (OVX) groups. **b** Volcano map showing the distribution of metabolites in the OVX + CEE-treated group vs. the OVX group. **c** Volcano map showing the distribution of metabolites in the OVX + TAM-treated group vs. OVX group

Approximately 30% of measured metabolites were statistically different across groups (Fig. 1 A–D). Volcano plots of detected biochemicals comparing metabolite profiles of tissue from Ovary-intact and OVX NHP identified 251 significantly upregulated and 15 downregulated metabolites (Fig. 1A). Volcano plots of detected biochemicals comparing metabolite profiles of tissue from OVX and OVX + CEE NHP identified 214 significantly upregulated and 19 significantly downregulated metabolites (Fig. 1B). Volcano plot of OVX and OVX + TAM tissue identified 70 significantly upregulated and 38 significantly downregulated metabolites (Fig. 1C).

### Carbohydrate metabolism

**Fig. 2:**
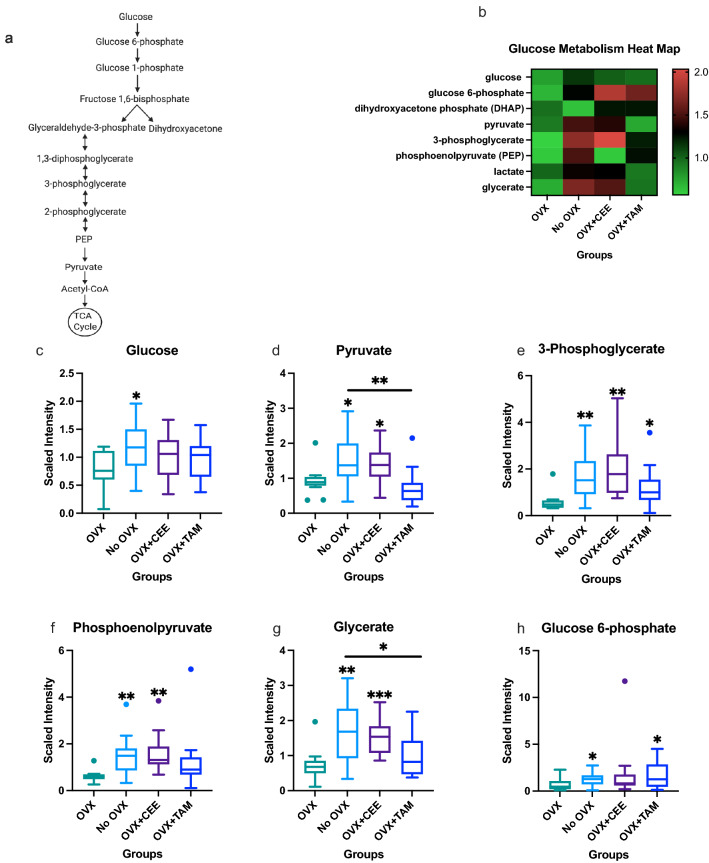
Changes in glucose metabolites associated with estrogen availability. **a** Glycolysis pathway. **b** Heat map showing effects of estrogens on glycolysis metabolites in mammary glands. **c** Differences in glucose metabolite across groups. **d** Differences in pyruvate metabolite across groups. **e** Differences in 3-phosphoglycerate across groups. **f** Differences in phosphoenolpyruvate across groups. **g** Differences in glycerate across groups. **h** Differences in glucose-6-phosphate across groups (*p* ≤ 0.05)

Changes in carbohydrate metabolism, specifically in glucose metabolism (Fig. 2), were regulated by menopause status and by HRT. Glucose metabolism (pathway shown in Fig. 2A) is critical to energy metabolism and mammary gland bioenergetics. Heat map in Fig. 2B shows all glucose metabolites measured in the NHP breast tissue. Samples from Ovary-intact NHP had elevated glucose levels compared with OVX NHP (Fig. 2C). Pyruvate was significantly elevated in ovary-intact and OVX + CEE NHP compared to OVX NHP (Fig. 2D). 3-phosphoglycerate levels were elevated in no-OVX, CEE- and TAM-treated NHP compared to OVX NHP (Fig. 2E).

Phosphoenolpyruvate levels were elevated in ovary-intact compared to OVX (Fig. 2F). CEE led to significant changes (*p* < 0.05) in phosphoenolpyruvate compared to OVX NHP (Fig. 2F). Ovary-intact and OVX + CEE NHP had elevated glycerate compared to OVX only (Fig. 2G). TAM treatment also had significant changes (*p* < 0.05) in glycerate compared to ovary-intact NHP (Fig. 2G). Glucose-6-phosphate was elevated in the ovary-intact NHP compared to the OVX NHP (Fig. 2H). TAM administration significantly increased glucose-6-phosphate levels compared to OVX tissue.

### Nucleotide sugar metabolism

**Fig. 3:**
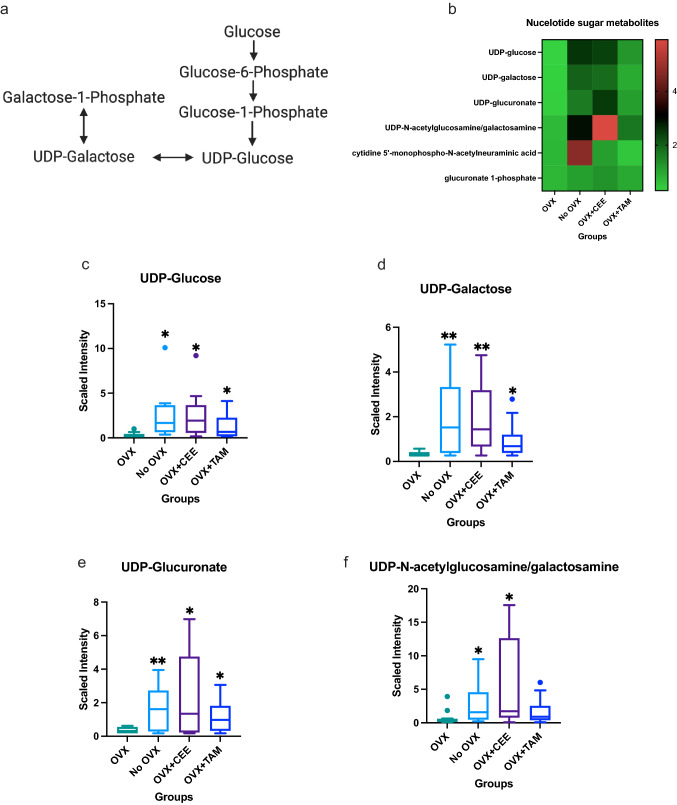
Changes in nucleotide metabolism mediated by estrogen. **a** Nucleotide metabolism pathway. **b** Heat map showing effects of estrogens on nucleotide sugar metabolites in mammary glands. **c** Differences in UDP-glucose across groups. **d** Differences in UDP-galactose across groups. **e** Differences in UDP-glucuronate across groups. **f** Differences in N-acetylglucosamine/galactosamine across groups (*p* ≤ 0.05)

Shifts in nucleotide sugar metabolism (Fig. 3) were noted with estrogen status (pathway shown in Fig. 3A) [49]. Heat map in Fig. 3B shows metabolites significantly regulated by ovariectomy and HRT administration. Specifically, UDP-glucose was elevated in ovary-intact compared to OVX NHP tissue (Fig. 3C). CEE and TAM treatment increased UDP-glucose (Fig. 3D). Ovary-intact NHP had elevated UDP-galactose compared to OVX tissue. CEE and TAM treatment also elevated UDP-galactose in OVX NHP compared to untreated OVX NHP (Fig. 3D); furthermore, the ovary-intact group had elevated UDP-glucuronate compared to the OVX group (Fig. 3E). CEE and TAM treatment increased UDP-glucuronate compared to OVX NHP (Fig. 3E). UDP-N-acetylglucosamine was elevated in the ovary-intact and OVX + CEE-treated NHP (Fig. 3F).

### Central energy metabolism

**Fig. 4:**
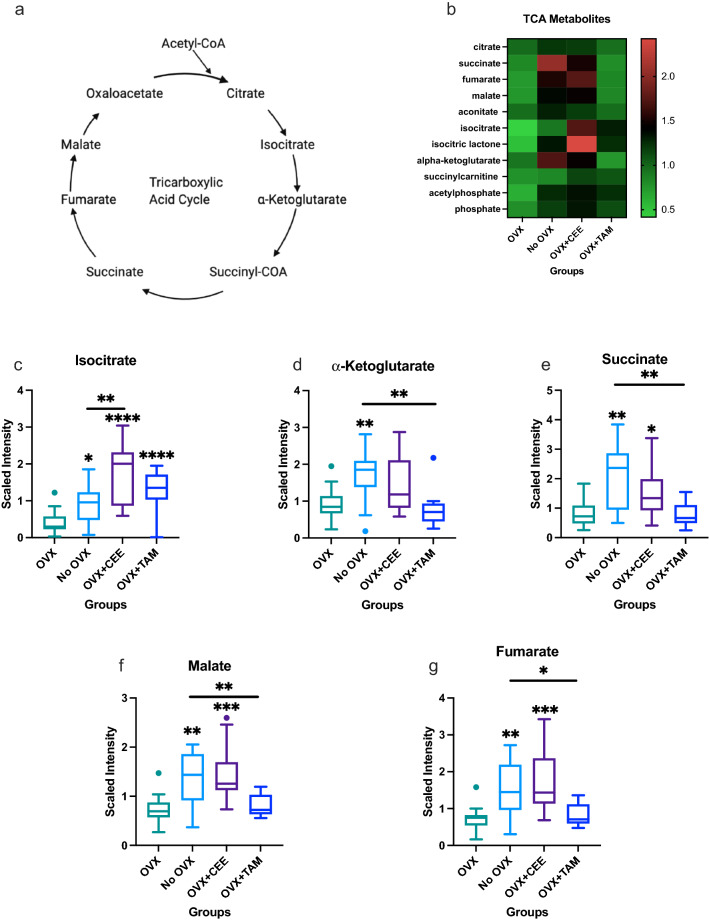
Changes in central energy metabolism associated with estrogen status. **a** Tricarboxylic acid cycle (TCA) pathway. **b** Heat map showing effects of estrogens on TCA metabolites in mammary glands. **c** Differences in isocitrate across groups. **d** Differences in α-ketoglutarate across groups. **e** Differences in succinate across groups. **f** Changes in malate across groups. **g** Changes in fumarate across groups. (*p* ≤ 0.05)

Alterations in central energy metabolism, including the TCA pathway shown in (Fig. 4A) and oxidative phosphorylation were observed (Fig. 4B). Isocitrate was elevated in ovary-intact NHP compared to OVX NHP (Fig. 4C). CEE and TAM treatment increased isocitrate compared to OVX-only NHP (Fig. 4C). Isocitrate was elevated in CEE-treated NHP compared to ovary-intact NHP (Fig. 4C). *α*-ketoglutarate was elevated in ovary-intact compared to OVX tissue. TAM treatment reduced *α*-ketoglutarate compared to ovary-intact NHP (Fig. 4D). Succinate was upregulated in ovary-intact and OVX + CEE-treated NHP compared to OVX NHP (Fig. 4E). TAM treatment displayed reduced succinate compared to the ovary-intact group (Fig. 4E). Ovary-intact and OVX + CEE NHP showed an increase in malate compared to OVX NHP (Fig. 4F). TAM treatment decreased malate compared to ovary-intact NHP breast tissue (Fig. 4F). Fumarate was elevated in the ovary-intact and OVX + CEE-treated tissue compared to the OVX NHP (Fig. 4G).

### Lipid metabolism

Perturbations in lipid metabolism, including fatty acid, phospholipid, bile acid, sterol, and corticosteroid metabolism, were observed. Individual regulated fatty acid metabolites are shown in a heat map (Supplemental Fig. S1A). Ovary-intact NHP tissue exhibited increases in acylcarnitines (Supplemental Figure S1A) and ketone body 3-hydroxy butyrate (BHBA; Supplemental Figure S1B) compared to the OVX group. CEE treatment increased arachidonoyl carnitine and linoleoyl carnitine (Supplemental Figure S1C & D). TAM treatment decreased fatty acid *β*-oxidation metabolites arachidonoyl carnitine and linoleoyl carnitine (Supplemental Figure S1C& D).

Changes in phospholipid biosynthesis metabolites were observed in breast samples from ovary-intact and OVX NHP (Supplemental Figure S2A). Among these metabolites were several phospholipid precursors, including choline phosphate, cytidine-5’-diphosphocholine, and cytidine-5’-diphosphoethanolamine (Supplemental Figure S2A). Notably, this increase in precursor molecules was also associated with changes in phosphatidylcholine (PC; Supplemental Figure S2B), phosphatidylethanolamine (PE; Supplemental Figure S2C), and phosphatidylserine (PS) species (Supplemental Figure S2D). Ovary-intact NHP had an increase in glycophosphoinositol compared to OVX NHP breast tissue (Supplemental Figure S2E). CEE-treated OVX NHP also displayed elevated glycophosphoinositol, similar to ovary-intact NHP (Supplemental Figure S2E). Choline phosphate was elevated in ovary-intact NHP compared to the OVX group (Supplemental Figure S2F). Additionally, CEE and TAM administration had increased choline phosphate compared to OVX NHP (Supplemental Figure S2F).

Significant changes in the cholesterol metabolites 7alpha-hydroxy-3-oxo-4-cholestenoate (7-Hoca) and 3beta-hydroxy-5-cholestenoate were also observed, as indicated by the heat map (Supplemental Figure S3A). CEE and TAM treatment reduced 7-HOCA compared to OVX only (Supplemental Figure S3B). 3β-hydroxy-5-cholestenoate levels were not significantly different between ovary-intact and OVX NHP breast tissue (Supplemental Figure S3C). Both CEE and TAM treatment reduced 3β-hydroxy-5-cholestenoate compared to the OVX-only NHP (Supplemental Figure S3C).

**Fig. 5:**
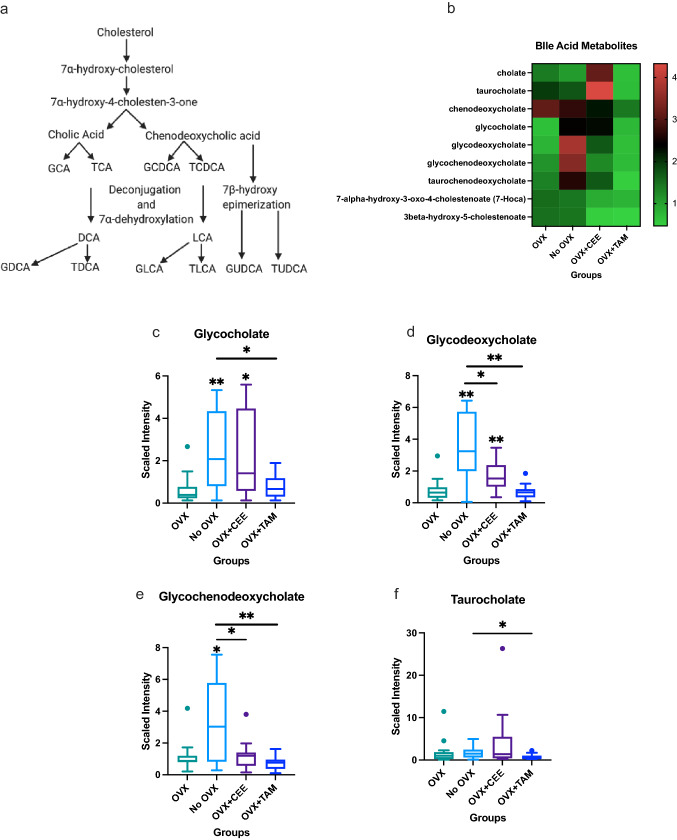
Changes in bile acid metabolism associated with estrogen bioavailability. **a** Bile acid synthesis pathway. **b** Heat map showing effects of estrogens on bile acid metabolism in mammary glands. **c** Changes in primary bile acid glycocholate metabolism across groups. **d** Changes in secondary bile acid glycodeoxycholate metabolism across groups. **e** Changes in secondary bile acid glycochenodeoxycholate metabolism across groups. **f** Changes in taurocholate across groups. (*p* < 0.05). (*GCA* glycocholic acid, *TCA* taurocholic acid, *GCDCA* glycochenodeoxycholic acid, *TCDCA* taurochenodeoxycholic acid, *DCA* deoxycholic acid, *LCA* lithocholic acid, *GDCA* glycodeoxycholic acid, *TDCA* taurodeoxycholic acid, *GLCA* glycolithocholic acid, *TLCA* taurolithocholic acid, *GUDCA* glycoursodeoxycholic acid, *TUDCA* tauroursodeoxycholic acid)

Primary bile acids are synthesized from cholesterol in hepatocytes, while secondary bile acids are formed by deconjugation and dihydroxylation reactions (Fig. 5A). Bile acid metabolism was altered by ovariectomy status and CEE and TAM administration (Fig. 5B). Significant increases in the primary bile acid glycocholate were observed in ovary-intact and OVX + CEE compared to OVX NHP (Fig. 5C). Glycocholate metabolism was decreased in OVX + TAM compared to ovary-intact NHP (Fig. 5C). Secondary bile acid glycodeoxycholate metabolism was also elevated in the ovary-intact and CEE-treated groups compared to OVX (Fig. 5D). The secondary bile acid glycochenodeoxycholate was also elevated in ovary-intact NHP (Fig. 5E). CEE and TAM reduced glycochenodeoxycholate compared to ovary-intact (Fig. 5E).

### Oxidative stress metabolism

**Fig. 6:**
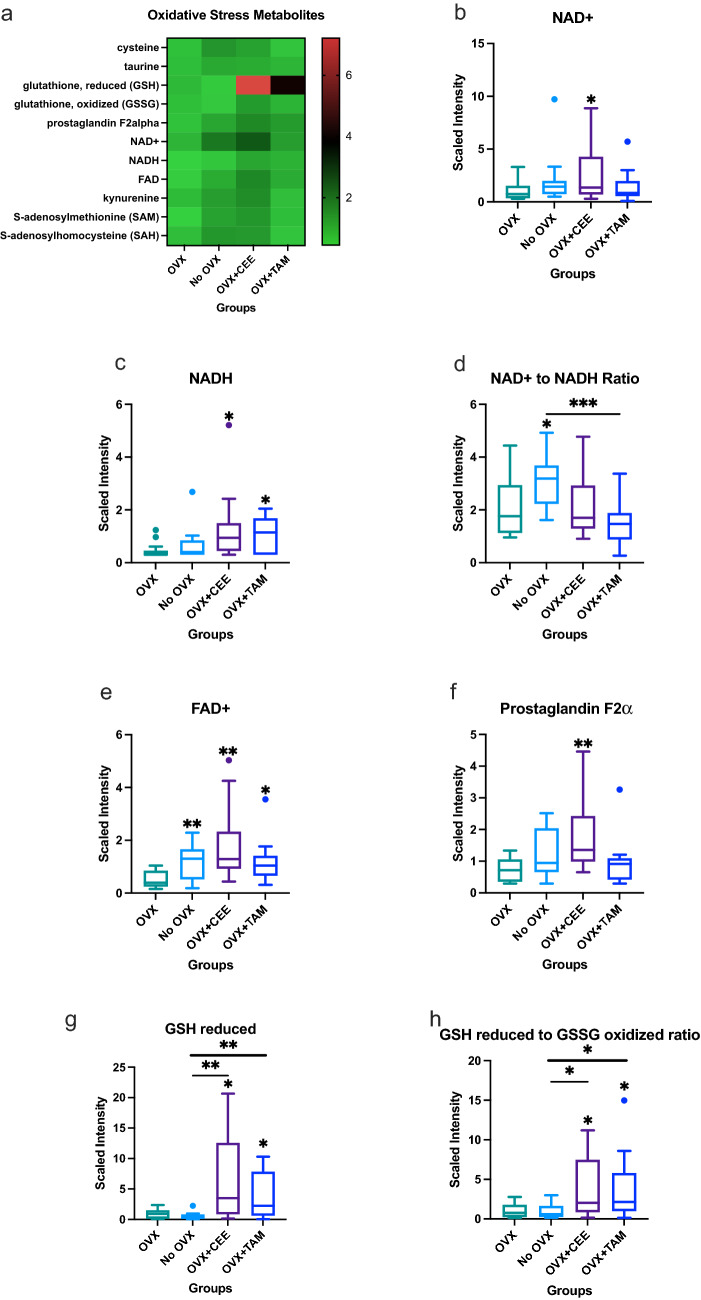
Changes in oxidative stress metabolites associated with estrogen status. **a** Heat map showing effects of estrogens on oxidative stress metabolites. **a** Changes in NAD + across groups. **c** Changes in NADH metabolism across groups. **d** Ratio of NAD + to NADH across groups. **e** Changes in FAD + across groups. **f** Changes in prostaglandin F2α across groups. **g** Changes in glutathione across groups. **h** Ratio of reduced to oxidized glutathione across groups

Alterations in several oxidative stress metabolites were observed (heat map; Fig. 6A). Increases in nicotinamide adenine dinucleotide (NAD +) were observed in the OVX + CEE NHP (Fig. 6B). NADH was not significantly different between the ovary-intact and OVX tissue; however, CEE significantly elevated NADH compared to the OVX-only NHP tissue (Fig. 6C). The ratio of NAD + to NADH was elevated in the ovary-intact NHP compared to the OVX NHP (Fig. 6D). FAD + was increased in the ovary-intact, OVX + CEE, and OVX + TAM breast tissue when compared to the OVX group (Fig. 6E). CEE also increased prostaglandin F2α (Fig. 6F). Reduced glutathione (GSH), an important antioxidant, was upregulated in CEE- and TAM-treated NHP compared to ovary-intact and OVX-only tissue (Fig. 6G). The ratio of reduced to oxidized glutathione, an important measure of oxidative stress, was significantly elevated in CEE- and TAM-treated NHP in relation to ovary-intact and OVX NHP (Fig. 6H).

## Discussion

Estrogen and estrogen metabolites play an important role in breast tissue homeostasis. Increased circulating E2 levels are associated with increased glucose uptake, directly linking estrogen to glucose metabolism [50]. Decreased glucose metabolism in the OVX group suggests that endogenous E2 promotes glycolysis in the mammary tissue. CEE increased all glycolytic intermediates as well as pyruvate in relation to the OVX group, suggesting that exogenous E2 administration restored glycolytic signaling comparable to ovary-intact breast levels. CEE treatment trended toward increasing glycolysis metabolites compared with the ovary-intact group, suggesting treatment with exogenous E2 might lead to overstimulation of glycolytic pathways in the mammary tissue.

Furthermore, studies have shown that E2 affects several glycolytic enzymes [51, 52]. Kostanyan and Nazaryan, which examined the effects of estradiol on several female rat brain glycolytic enzymes, showed that hexokinase, phosphofructokinase, and pyruvate kinase were elevated 4 h after treatment [51]. Conversely, limited glucose changes were noted in the TAM-treated group compared with OVX tissue. Tamoxifen increased glucose-6-phosphate and 3-phosphoglycerate compared to OVX tissue. Our study in noncancerous OVX NHP indicates that low estrogen bioavailability (modeling menopause), decreased normal breast tissue glycolysis. Tamoxifen led to a modest increase of these regulated metabolites, suggesting a partial restoration of glucose metabolism to ovary-intact levels. Overall, these results suggest that endogenous (ovary-intact) and exogenous (CEE administration) estrogen might promote glycolysis in mammary tissue to maintain normal tissue bioenergetic homeostasis. Moreover, that tamoxifen-mediated regulation of bioenergetics pathways may represent, in part, the drug’s cancer prevention mechanism of action.

Nucleotide sugars act as glycosyl donors. Glycosylation of cell surface proteins mediates numerous aspects of cell behavior, including cell–cell communication, adhesion, and migration [53]. Glycosylation of intracellular proteins mediates signal transduction and gene regulation [54, 55]. We showed CEE treatment increased UDP-glucuronate metabolism in OVX NHP, suggesting estrogen may be related to breast cancer by mediating aberrant nucleotide sugar metabolism. UDP-*N*-acetylglucosamine (UDP-GlcNAc), which was decreased in OVX NHP but elevated with CEE treatment, has been correlated with breast cancer. UDP-GlcNAc, produced by fructose-6-phosphate, is a precursor in hyaluronan production. Hyaluronan is involved in inflammation [56], invasion, metastasis [57], and epithelial to mesenchymal transition (EMT; [58]). Hyaluronan is elevated in numerous tumor types, including breast tumors, and is associated with poor patient survival [57, 59].

Endogenous estrogens control energy homeostasis by regulating appetite, adiposity, and increasing energy expenditure [60–62]. Consistently, significant increases in TCA metabolites were noted in the ovary-intact compared to the OVX group, supporting increased energy expenditure due to endogenous estrogen. Exogenous estrogens may promote energy homeostasis by influencing energy expenditure. As with the ovary-intact group, CEE showed increased TCA metabolites, suggesting exogenous estrogens exert effects similar to endogenous estrogens on energy expenditure. Specifically, isocitrate was elevated above ovary-intact levels with CEE treatment, suggesting exogenous estrogens may enhance TCA metabolites past endogenous E2 regulation. However, limited changes in energy metabolism were observed in the TAM-treated NHP compared to the untreated OVX NHP, suggesting estrogen receptor blockade does not mediate further changes in TCA metabolism in normal breast tissue with low estrogen bioavailability. Overall, the changes in the mammary tissue’s energy metabolism are due to estrogen-induced changes.

Increases in acylcarnitine species in the ovary-intact group suggest lipid oversupply and upregulated fatty acid oxidation [63], as estrogen increases fatty acid utilization and oxidation [64, 65]. Increases in the ketone 3-hydroxy-butyrate (BHBA), the metabolic fuel in oxidative phosphorylation, suggests changes in fatty acid mobilization and *β*-oxidation in the ovary-intact group [66]. Furthermore, increases in phospholipids suggest membrane remodeling and/or repair of damaged tissue membranes, consistent with the protective effects of estrogen on cellular structures [38]. TAM treatment in the NHP decreased both fatty acid metabolites and cholesterol metabolites, consistent with its actions as an estrogenic agonist on lipid metabolism.

Estrogen affects bile acid (BA) synthesis by influencing both enzymatic activity and BA pool composition. For example, transfection of human embryonic kidney (HEK) 293 cells with ERα and ERβ with estrogen treatment upregulates expression of CYP7B1, an enzyme that hydroxylates cholesterol into the bile acid chenodeoxycholic acid [24]. Treatment of rats with ethynylestradiol, a common estrogenic component of oral contraceptives, increased the relative amount of bile acids [67]. However, while CEE elevated bile acid levels above ovariectomized levels, these levels were significantly lower than ovary-intact levels. Suggesting endogenous and exogenous estrogens differentially effect bile acid synthesis.

Chronic inflammation is associated with breast cancer [68]. Elevated reactive oxygen species (ROS) contribute to inflammation and promote tumor development and progression by causing DNA damage, increasing the mutation rate within cells, promoting oncogenic activity [69]. In cancer cells, increased metabolic activity, mitochondrial dysfunction, and oncogene activity all result in high levels of ROS [69–72]. Prostaglandin F2 α (PGF2α), an important inflammatory molecule, is elevated in women with breast cancer compared to healthy controls. However, high PGF2α in tumors is associated with good prognosis compared to tumors with low levels of PGF2α [73]. Estrogen increases the production of PGF2α, consistent with increases observed in the CEE group, suggesting treatment with CEE may confer a better tumor prognosis [74].

Reduced glutathione (GSH), which scavenges reactive oxygen species, was elevated in the CEE-treated group [75]. The ratio of reduced glutathione to oxidized glutathione (GSSG) was also increased in the CEE-treated group. This ratio serves as a measure of oxidative damage, with a high ratio of GSH to GSSG associated with reduced oxidative damage as GSSG is associated with injury and oxidative stress [46]. A reduction in ROS production and inhibition of inflammation may prevent breast carcinogenesis [45].

Estrogen promotes breast epithelium proliferation. Breast proliferation and gland epithelium percentage in the NHP was assessed by Cline, Soderqvist et al. 1998. Results indicated a small, but significant increase in epithelium content in OVX + CEE-treated NHP compared with OVX NHP. There was no significant difference in epithelium content in breast tissue samples between OVX + TAM and OVX NHP. Therefore, while the alterations in metabolites measured between comparisons are most likely not due to variations in epithelial cell content in breast tissue, it should be noted as a potential limitation to the study [44].

Overall, the results of this study suggest that estrogen bioavailability is associated with estrogen-dependent changes in the mammary tissue metabolome, particularly in glucose and fatty acid metabolism. Changes in these pathways provide a link between estrogen action and energy homeostasis. Further study is needed to determine how metabolic changes associated with estrogen availability affect health and disease states, particularly breast cancer, in premenopausal and postmenopausal individuals. Further studies on the actions of different hormone replacement therapies, such as aromatase inhibitors, on metabolites is needed.

## Supplementary Information

Below is the link to the electronic supplementary material.

Supplementary file1 (PDF 37 KB)

Supplementary file2 (PDF 49 KB)

Supplementary file3 (PDF 37 KB)

Supplementary file4 (PDF 121 KB)

Supplementary file5 (DOCX 29 KB)

Supplementary file6 (XLSX 4695 KB)

## Data Availability

Spreadsheet of raw and processed metabolomics data generated from Metabolon are available as supplemental data associated with the manuscript.

## Declarations

### Research involving Human and Animal participants

All animal experimental protocols were approved by the Wake Forest University Institutional Animal Care and Use Committee.
