# Supplementary figures and images for: Investigating the role of endogenous estrogens, hormone replacement therapy, and blockade of estrogen receptor-α activity on breast metabolic signaling
Source: Breast Cancer Res Treat. 2021 Aug 26;190(1):53–67. doi: 10.1007/s10549-021-06354-w (PMC8557185; doi:10.1007/s10549-021-06354-w)

a

## Fatty Acid Metabolites

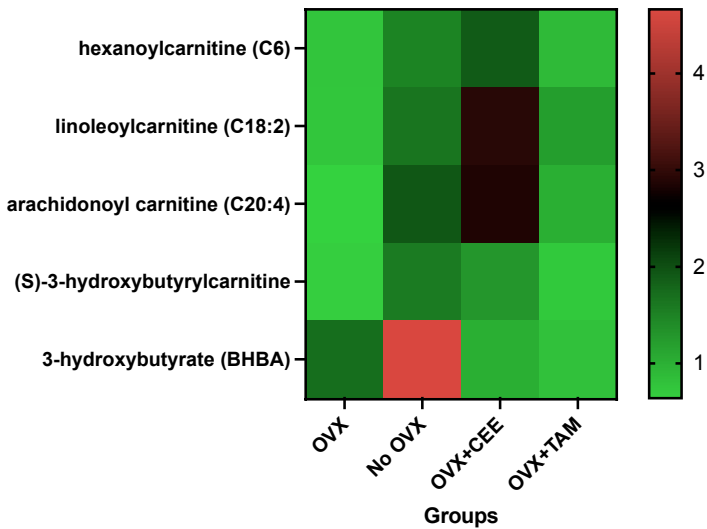

b

## 3-hydroxybutyrate

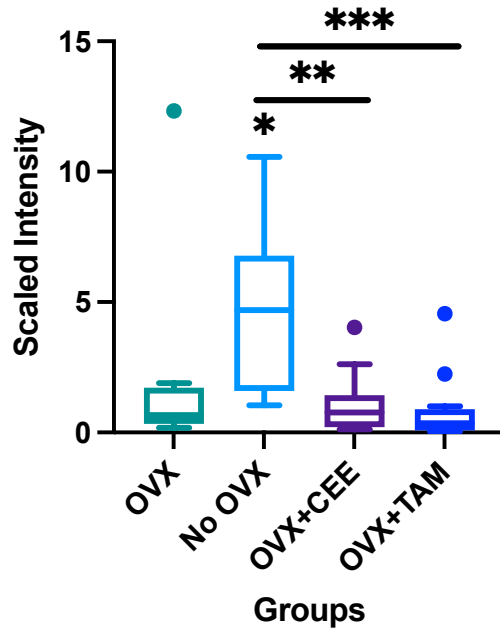

Supplement: Supplementary file 1 — Supplementary file1 (PDF 37 KB) [file 10549_2021_6354_MOESM1_ESM.pdf]

a

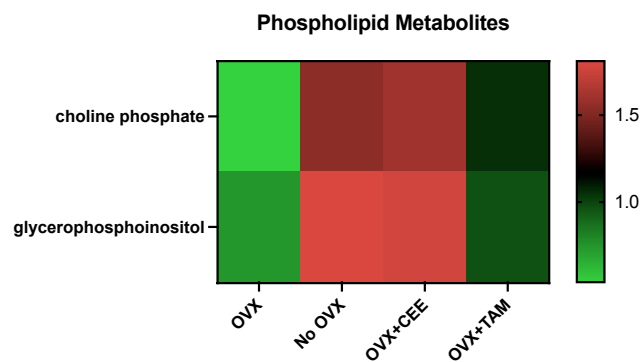

b

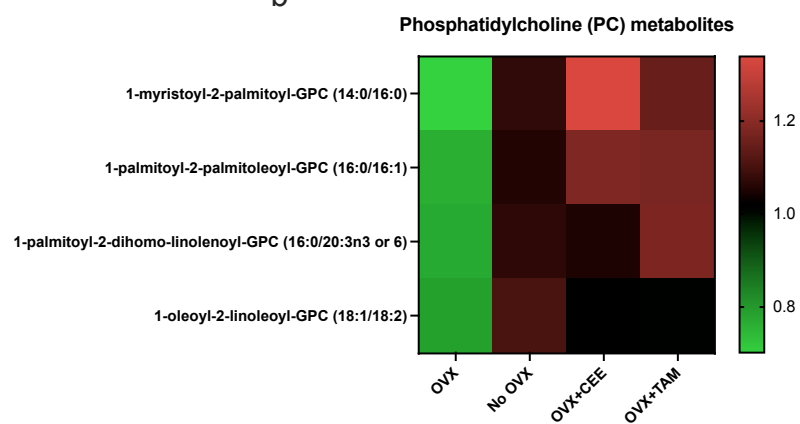

c

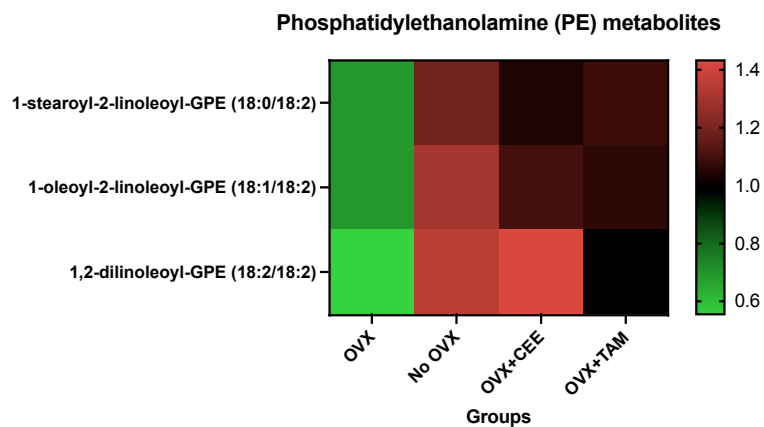

d

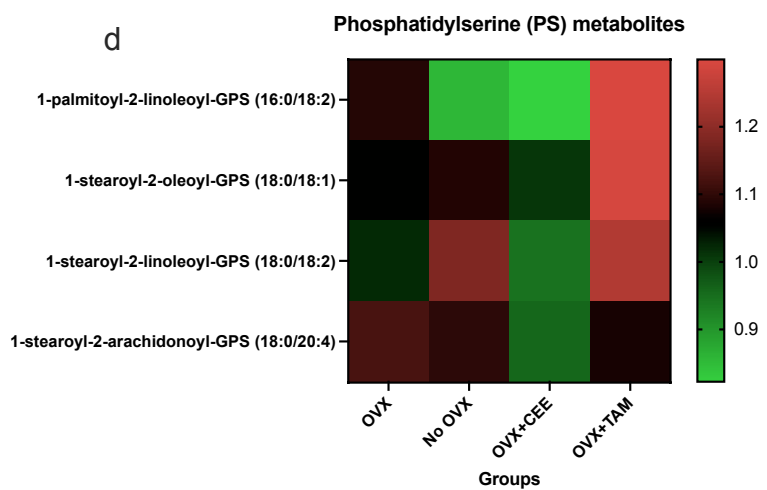

e

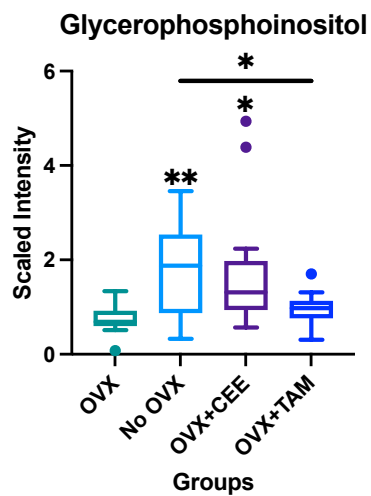

f

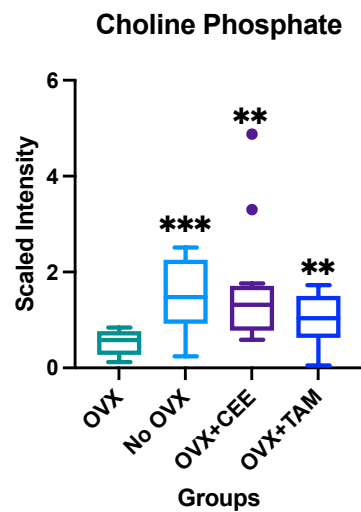

Supplement: Supplementary file 2 — Supplementary file2 (PDF 49 KB) [file 10549_2021_6354_MOESM2_ESM.pdf]

a

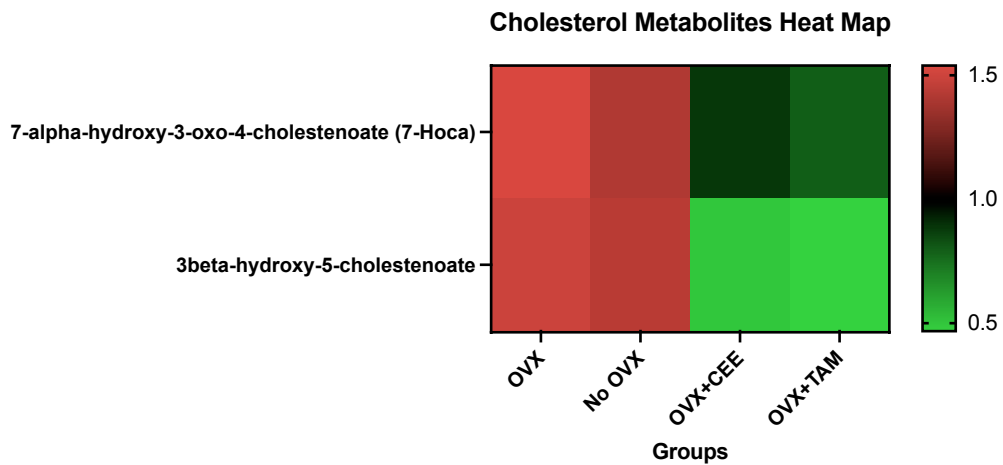

b

7-Hoca

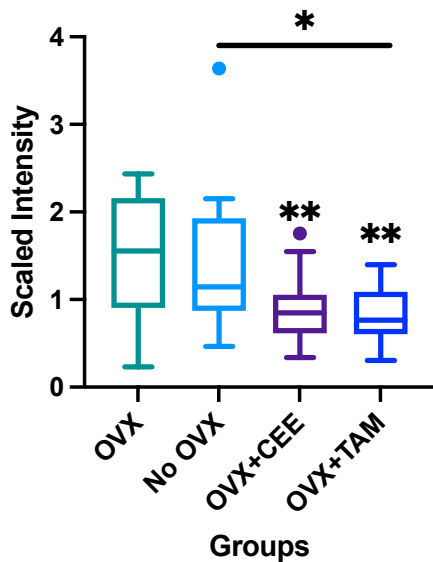

c

3beta-hydroxy-5-cholestenoate

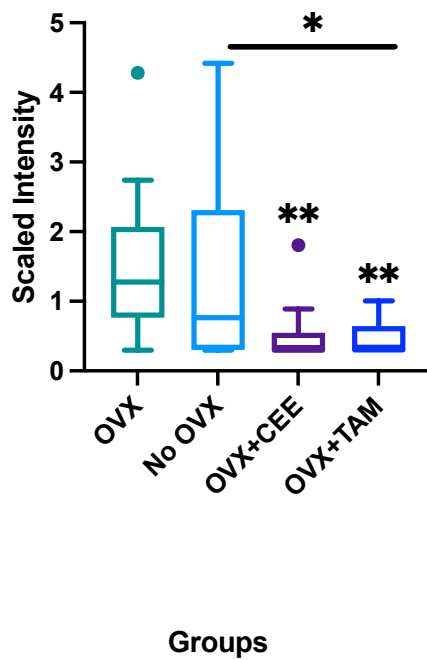

Supplement: Supplementary file 3 — Supplementary file3 (PDF 37 KB) [file 10549_2021_6354_MOESM3_ESM.pdf]

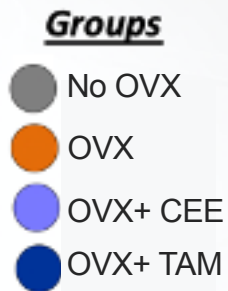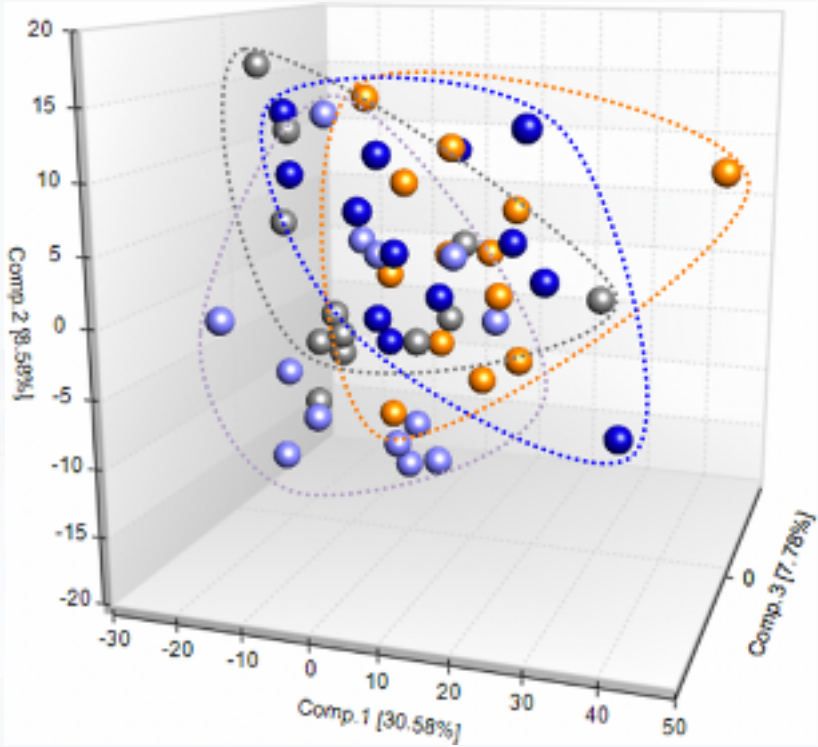

Supplement: Supplementary file 4 — Supplementary file4 (PDF 121 KB) [file 10549_2021_6354_MOESM4_ESM.pdf]
