# Supplementary material for: Investigating the role of endogenous estrogens, hormone replacement therapy, and blockade of estrogen receptor-α activity on breast metabolic signaling
Source: Breast Cancer Res Treat. 2021 Aug 26;190(1):53–67. doi: 10.1007/s10549-021-06354-w (PMC8557185; doi:10.1007/s10549-021-06354-w)
Supplement: Supplementary file 5 — Supplementary file5 (DOCX 29 KB) [file 10549_2021_6354_MOESM5_ESM.docx]

**Supplemental Material:**

**Supplemental Table 1**: Summary of the number of biochemicals that reached statistical significance (p<0.05) or those that approached significance (0.05<p<0.10) across groups.

**Supplemental Figure Legends**:

Supplemental Figure 1: Changes in fatty acid metabolism with estrogen ablation. (A) Heat map showing effects of estrogens on fatty acid metabolites in mammary glands. (B) Differences in BHBA across groups. (p<0.05).

Supplemental Figure 2: Changes in membrane phospholipid metabolites. (A) Heat map showing differences in phospholipids across groups. (B) Heat map showing differences in the phosphatidylethanolamine species. (C) Heat map showing changes in phosphatidylcholine species. (D) Heat map showing changes in phosphatidylserine species. (E) Differences in glycophosphoinositol across groups. (F) Differences in choline phosphate across groups. (p<0.05)

Supplemental Figure 3: Changes in cholesterol metabolites. (A) Heat map showing effects of estrogen on cholesterol metabolites in mammary glands. (B) Differences in 7-alpha-hydroxy-3-oxo-4-cholestenoate (7-HOCA) metabolism across groups (p<0.05).

Supplemental Figure 4: Principal component analysis segregating mammary gland samples based on estrogen availability status (Ovary-intact (no-OVX) vs Ovariectomized (OVX)) and/or treatment after OVX (Untreated, CEE, or TAM). The samples displayed minimal separation based on both estrogen status and treatment post menopause in the PCA.
